# Expiratory Muscle Relaxation-Induced Ventilator Triggering: A Novel Patient-Ventilator Dyssynchrony

**DOI:** 10.1016/j.chest.2022.01.070

**Published:** 2022-06-06

**Authors:** Annemijn H. Jonkman, Minke C. Holleboom, Heder J. de Vries, Marijn Vriends, Pieter R. Tuinman, Leo M.A. Heunks

**Affiliations:** a Department of Intensive Care Medicine, Amsterdam University Medical Center, location VUmc, Amsterdam, The Netherlands; b Department of Intensive Care Medicine, Erasmus Medical Center, Rotterdam, The Netherlands

**Keywords:** expiratory muscles, mechanical ventilation, patient-ventilator dyssynchrony

## Abstract

In critically ill patients receiving mechanical ventilation, expiratory muscles are recruited with high respiratory loading and/or low inspiratory muscle capacity. In this case report, we describe a previously unrecognized patient-ventilator dyssynchrony characterized by ventilator triggering by expiratory muscle relaxation, an observation that we termed *expiratory muscle relaxation-induced ventilator triggering* (ERIT). ERIT can be recognized with in-depth respiratory muscle monitoring as (1) an increase in gastric pressure (Pga) during expiration, resulting from expiratory muscle recruitment; (2) a drop in Pga (and hence, esophageal pressure) at the time of ventilator triggering; and (3) diaphragm electrical activity onset occurring after ventilator triggering. Future studies should focus on the incidence of ERIT and the impact in the patient receiving mechanical ventilation.

In critically ill patients receiving mechanical ventilation, expiratory muscles are recruited with high respiratory loading and/or low inspiratory muscle capacity, or with pulmonary hyperinflation.^1^, ^2^, ^3^ Critical illness is associated with time-dependent changes in expiratory muscle mass,^4^ and coordination of activation between inspiratory muscles and expiratory muscles in ventilated patients may be disturbed.^2^ Herein, we present a previously unrecognized patient-ventilator dyssynchrony characterized by ventilator triggering by expiratory muscle relaxation.

## Case Report

A patient (50 years of age; BMI, 43 kg/m^2^) with no relevant medical history was intubated endotracheally because of COVID-19 respiratory failure. During pressure support ventilation, expiratory muscle contractions were recognized by visual observation and abdominal palpation. To evaluate respiratory muscle mechanics, we inserted a double-balloon nasogastric catheter (Sidam) and an electrical activity of the diaphragm (EAdi) catheter (Getinge) and performed analyses while varying the pressure support level (range, 16-2 cm H_2_O) within a 30-min period.

**Figure 1:**
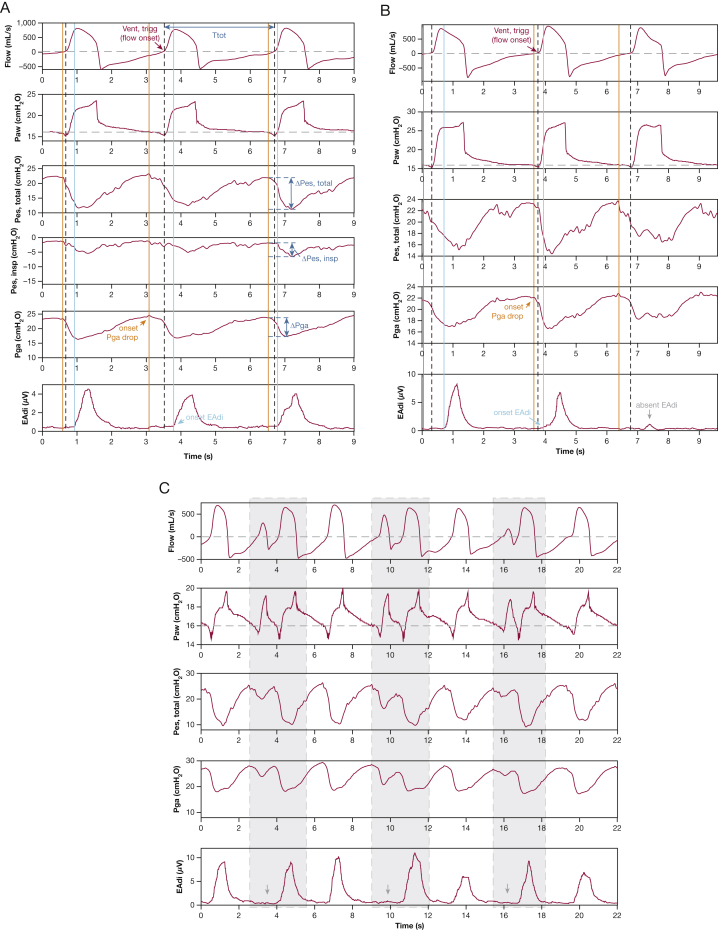
A-C, Demonstration of expiratory muscle relaxation-induced ventilator triggering (ERIT). Flow, Paw, Pes,total (ie, uncorrected Pes signal), Pes,insp (ie, Pes,total – Pga), Pga, and EAdi waveforms recorded during pressure support ventilation with 6 cm H_2_O (A), 10 cm H_2_O (B), and 2 cm H_2_O (C) of support above a positive end-expiratory pressure of 16 cm H_2_O. Pao_2_ to Fio_2_ ratio was 132 mm Hg, and the patient was receiving continuous sedation (Richmond Agitation Sedation Scale score of –4, with propofol 6.6 mg/kg predicted body weight/h and fentanyl 0.66 μg/kg predicted body weight/h). Before recording, adequate position of the double-balloon catheter and EAdi catheter was confirmed using the Baydur maneuver and esophageal spasms present in the Pes tracing, but not in the Pga tracing, and by using the EAdi positioning tool provided on the Servo-U ventilator (Getinge), respectively. Ventilator flow, Paw, and EAdi waveforms were obtained from the ventilator using Servo-tracker software (release 4.2). Simultaneously, Paw, Pes, and Pga were acquired with a dedicated measurement setup (Biopac MP160; BIOPAC, Inc.). Data were synchronized based on Paw tracings that were acquired simultaneously using both acquisition devices and were processed using a software routine for MATLAB 2020b (Mathworks). The onset for different signals was defined as follows (A, B): (1) onset ventilator triggering (Vent,trigg; black dashed line) was defined as start of inspiratory flow (ie, nadir in Paw); (2) onset expiratory muscle relaxation (orange solid line) was defined as start of continuous decrease in Pga; and (3) onset EAdi (light blue solid line) was defined as start of inspiratory EAdi increase of > 0.5 μV, provided that EAdi peak is > 2 μV (to qualify as a breath). Ttot was defined based on flow zero-crossings (A, blue arrow in flow signal). Total Pes decrease was calculated from the onset in Pga drop (concomitant with start of decrease in Pes,total) to Pes,total nadir. The decrease in Pes,insp (ie, reflecting the patient's true inspiratory effort) was calculated from the onset in Pga drop (concomitant with start of decrease in Pes,insp) to Pes,insp nadir. The Pga drop from expiratory muscle relaxation was calculated from the onset in Pga drop to Pga nadir. A, EAdi onset started after ventilator triggering, whereas the drop in Pga and Pes occurred before ventilator triggering. The start of drop in Pga and Pes was very close to the first time point of the sudden decrease in Paw below set positive end-expiratory pressure (start of triggering phase). B, Example showing that not all ERIT breaths were followed by inspiratory effort (arrow): EAdi < 2 μV and the total Pes decrease was approximately equal to the Pga drop from expiratory muscle relaxation. C, Example of double breaths (gray area) with ERIT in a 2:1 pattern. The first ventilator pressurization was the result of some degree of expiratory muscle relaxation not followed by patient inspiratory effort (absent EAdi indicated by arrow in EAdi signal and the total Pes decrease approximately equal to the Pga drop). Then, complete expiratory muscle relaxation resulted in a ventilator insufflation followed by an inspiratory effort. EAdi = electrical activity of the diaphragm; Paw = airway pressure; Pes = esophageal pressure; Pes,insp = esophageal pressure related to inspiratory effort; Pes,total = total esophageal pressure; Pga = gastric pressure; Ttot = total ventilator cycle duration; Vent,trigg = ventilator triggering.

A remarkable patient-ventilator interaction was observed, which we termed *expiratory muscle relaxation-induced ventilator triggering* (ERIT) (Fig 1). EAdi onset occurred after ventilator triggering, indicating that the ventilator was not triggered by the diaphragm. The esophageal pressure (Pes) drop just before ventilator triggering may indicate triggering with extradiaphragmatic inspiratory muscles. However, a concomitant decrease in gastric pressure (Pga) was observed after the Pga rise during the preceding expiration. Thus, ventilator triggering resulted from expiratory muscle relaxation.

To quantify the timing of respiratory muscle activity in relationship to ventilator triggering (Vent,trigg), we calculated the phase angle (PA), a parameter that also has been used for characterizing reverse triggering, for instance.^5^ PA between the Pga drop and Vent,trigg was defined as:

$${\text{PA}}_{\text{Pga}\;\text{drop}\;-\;\text{Vent,trigg}}\;=\;\left(\left[\text{onset}\;\text{time}\;\text{Pga}\;\text{drop}\;-\;\text{inspiratory}\;\text{flow}\;\text{onset}\right]/\text{Ttot}\right)\;\times \;360{}^{\circ}$$

where Ttot is the ventilator cycle duration. To qualify as ERIT, PA_Pga drop –_ _Vent,trigg_ should be < 0°, and PA between EAdi onset and triggering (PA_EAdi onset – Vent,trigg_) should be > 0°. We also quantified inspiratory effort with EAdi_peak_ and Pes. The Pes,total decrease is determined by the Pga drop from expiratory muscle relaxation plus the patient's true inspiratory effort (Pes,insp); therefore, Pes,insp can be computed as Pes,total – Pga (Fig 1A).

For a 10-breaths period (Fig 1A), median PA_Pga drop – Vent,trigg_ was –38.7° (interquartile range [IQR], –45.5° to –29.7°), median PA_EAdi onset – Vent,trigg_ was 15.4° (IQR, 12.8°-18.6°), median Pga drop was 8.0 cm H_2_O (IQR, 7.6-8.3 cm H_2_O), and median total Pes decrease was 11.0 cm H_2_O (IQR, 10.8-11.5 cm H_2_O); only 3.8 cm H_2_O (IQR, 3.4-4.2 cm H_2_O) reflected the Pes,insp. Median EAdi_peak_ was 3.7 μV (IQR, 3.3-4.1 μV).

ERIT was present at all applied pressure support levels. Notably, in some ERIT breaths, inspiratory muscle activity was negligible (Fig 1B) (total Pes decrease is approximately equal to the Pga drop from expiratory muscle relaxation, with almost absent EAdi). Occasionally, a pattern of double breaths was observed (Fig 1C): the first ventilator triggering resulted from partial expiratory muscle relaxation not followed by inspiratory effort. Then, complete expiratory muscle relaxation resulted in ventilator triggering followed by inspiratory effort. Double breaths seemed to occur in a specific pattern; ratios of 2:1 (Fig 1C) and 3:1 were observed in the patient.

**Figure 2:**
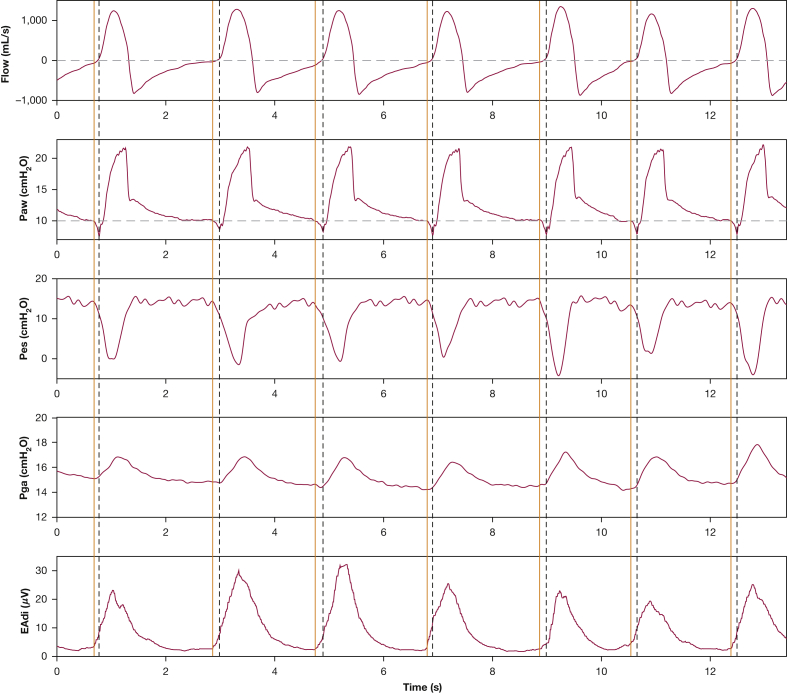
Observations obtained 5 days after the recording of Figure 1 (pressure support, 8 cm H_2_O; positive end-expiratory pressure, 10 cm H_2_O; respiratory rate, 34 breaths/min; tidal volume, 400-470 mL [5.5-6.3 mL/kg predicted body weight]; and Pao_2_ to Fio_2_ ratio, 159 mm Hg under continuous sedation [Richmond Agitation Sedation Scale score, –3; propofol, 2.5 mg/kg predicted body weight/h; fentanyl, 1.33 μg/kg predicted body weight/h]). Patient effort was perfectly synchronous with the ventilator: the onset of Pes decrease and EAdi increase occurred at the same time (orange solid line) and before ventilator triggering (black dashed line) for each breath. No signs of expiratory muscle recruitment (no increase in Pga during expiration) were found. EAdi = electrical activity of the diaphragm; Paw = airway pressure; Pes = esophageal pressure; Pga = gastric pressure.

The patient was reassessed 5 days later. Inspiratory effort was perfectly synchronous with the ventilator (Fig 2), and no expiratory muscle recruitment was observed. The fact that diaphragm neuromechanical efficiency was similar to that 5 days earlier indicated that diaphragm function remained stable.

## Discussion

To our knowledge, this is the first description of ventilator triggering by expiratory muscle relaxation (ERIT). This dyssynchrony is characterized by: (1) an increase in Pga during expiration, resulting from expiratory muscle recruitment; (2) a drop in Pga (and hence, Pes) at the time of ventilator triggering; and (3) EAdi onset occurring after ventilator triggering. Visual observation, palpation, and ultrasound could screen for expiratory muscle recruitment,^3^^,^^6^ but in-depth waveform analyses are needed to identify ERIT. This stresses the importance of monitoring the complex interplay between the inspiratory and expiratory muscles during mechanical ventilation. Because the decrease in both Pes and Pga coincided, manometry alone cannot identify ERIT, and EAdi should be recorded simultaneously. Moreover, both Pga and Pes are required to distinguish between triggering resulting from extradiaphragmatic inspiratory muscles and expiratory muscles. Although expiratory muscles frequently are recruited in the critically ill,^1^, ^2^, ^3^ affect intrinsic positive end-expiratory pressure measurement, and may even contribute to weaning failure,^7^ the complexity of recognizing ERIT could explain why this type of dyssynchrony has not been described before. Clinical and physiologic consequences of ERIT remain uncertain; however, not recognizing expiratory muscle recruitment may affect inspiratory effort calculation (ie, overestimate) using Pes. Future studies should focus on the incidence of ERIT, potential solutions to resolve this dyssynchrony, and the clinical impact in patients receiving mechanical ventilation.
